# Scanning Micromirror Platform Based on MEMS Technology for Medical Application

**DOI:** 10.3390/mi7020024

**Published:** 2016-02-06

**Authors:** Eakkachai Pengwang, Kanty Rabenorosoa, Micky Rakotondrabe, Nicolas Andreff

**Affiliations:** 1 Automatic Control and Micro-Mechatronic Systems Department (AS2M), FEMTO-ST Institute, UMR CNRS 6174-UFC/ENSMM/UTBM, Besancon 25000, France; kanty.rabenorosoa@femto-st.fr (K.R.); micky.rakotondrabe@femto-st.fr (M.R.); nicolas.andreff@femto-st.fr (N.A.); 2 Institute of Field Robotics, King Mongkut’s University of Technology Thonburi, 126 Pracha Uthit Road, Bang Mod, Thung Khru, Bangkok 10140, Thailand

**Keywords:** micro robots, optical MEMS, MEMS scanning micromirror, biomedical micro-actuator, multi-degree-of-freedom stage, bioMEMS, MOEMS, micro-optics

## Abstract

This topical review discusses recent development and trends on scanning micromirrors for biomedical applications. This also includes a biomedical micro robot for precise manipulations in a limited volume. The characteristics of medical scanning micromirror are explained in general with the fundamental of microelectromechanical systems (MEMS) for fabrication processes. Along with the explanations of mechanism and design, the principle of actuation are provided for general readers. In this review, several testing methodology and examples are described based on many types of actuators, such as, electrothermal actuators, electrostatic actuators, electromagnetic actuators, pneumatic actuators, and shape memory alloy. Moreover, this review provides description of the key fabrication processes and common materials in order to be a basic guideline for selecting micro-actuators. With recent developments on scanning micromirrors, performances of biomedical application are enhanced for higher resolution, high accuracy, and high dexterity. With further developments on integrations and control schemes, MEMS-based scanning micromirrors would be able to achieve a better performance for medical applications due to small size, ease in microfabrication, mass production, high scanning speed, low power consumption, mechanical stable, and integration compatibility.

## 1. Introduction

Scanning micromirrors play an important role in modern technology. It involves several principles on many related fields of research, such as micropositioning, micromachining, microscopy, precision measurements, and imprint lithography. Commercial and industrial uses of micromirrors can be found in optical devices, tunable lasers [1], televisions, hard disk storage, projectors, and imaging technologies [2]. The history of scanning micromirrors or digital micromirror device (DMD) began in the year of AD 1987 for an application of digital light projectors (DLP), digital cinema, home entertainment sets, and optomechanical components. The concepts of digital micromirrors are the device that can create images by altering different light sources. The mechanical design of this micromirrors is a silicon micromirrors with a pair of torsion hinges. The capabilities of optical scanning angle of this type are within a range of ±10°. Although the market values of this digital light processing micromirrors are growing for a few decades, the development of micromirrors with advanced techniques is still in progress for higher ranges of motion, faster speed, multi-directions, and broader their applications.

For medical applications, scanning micromirrors have been developed for a submicron biomedical system, such as, optical scanning, microscopy, confocal microscopy, medical endoscopy, laparoscopy, and optical coherence tomography (OCT) [3]. In general, the principal of scanning micromirrors is still the same with the previous technologies; altering the light sources for surgery, scanning the targeted areas, and collecting the bioimaging from the surgical locations. Therefore, design of scanning micromirrors has to be small in volume, inexpensive, and compatible with fiber optic systems [4]. These characteristics are suitable for collecting OCT images of internal architectural morphology and cellular structures in the tissue. Examples of implemented areas include gastrointestinal tract, esophageal, gastric, colonic mucosa, colonic adenoma, respiratory tracts, and carcinoma. While a conventional processes such as computed tomography (CT), positron emission tomography (PET), ultrasound, and magnetic resonance imaging (MRI), can provide a resolution in a range of 100 μm, the optical coherence tomography with micromirrors is reported for a resolution in a range of 10 μm. Indeed, *in vivo* endoscopic OCT can provide a high penetration depth and high resolution images [4,5]. By implementing an optical coherence reflectometry for a broadband light source, OCT is reported to be a nondestructive, high resolution, and minimally invasive real time imaging method. The method of scanning for OCT can be either linear or rotational. With a further development of signal analysis and noise reduction, OCT can achieved a high speed scanning and high dynamic range for both two-dimensional (2D) and three-dimensional (3D) imaging. This OCT method can be used for cross-sectional imaging for medical, biopsy, and biophotonic applications. However, main challenge of endoscopic OCT is a reliable and accessible of probing low-coherence radiation to the surface of internal organs. In common, flexible fiber optic bundles have been used for endoscopic OCT to access the surgical areas and delivery a light source for surgery. Therefore, the design of scanning micromirrors, distal end, catheter, and the integration of endoscopic OCT needs be done carefully.

Moreover, the development of these scanning micromirrors will enhance capabilities of medical robots for minimally invasive soft tissue surgery, neurosurgery, ear nose and throat (ENT) surgery, phonosurgery, thoracic surgery, cardiac surgery, respiratory tracts surgery, and urologic surgery [6]. A scanning micromirror also improves the development of laser incision processes and skills of physicians. In general, developments of OCT and minimally invasive surgeries (MIS) systems require scanning micromirrors with high resolution, high accuracy, high dexterity, while the dimensions are limited. In common, MIS will involve with a small incision that are enough for fiber optic, endoscopy, and surgical tools. This surgical processes with small incision will result to a faster recovery of patients, less trauma to the body, less blood loss, reduced length of hospital stay. This technique can be enabled by the development of laser surgery and endoscopic imaging by using a scanning micromirror.

Due to the integration constraints of scanning micromirrors, their design, modeling and fabrication have been investigated for the past decades in order to miniaturize and improve the performances. Many successful medical applications have been reported. Common processes to manufacture scanning micromirrors are microelectromechanical systems (MEMS) technology because the processes can create submicron features with high precision, mass productive, and low cost per unit. MEMS-based processes is also suitable for creating biomedical apparatus that require high speed, low power consumption, and high reliability. Several microfabrication techniques are implemented to create scanning micromirrors and their apparatus. Both additive and subtractive processes can be used for the microfabrication of scanning micromirrors. Common substrates can be silicon, glass, thin film of metals, photoresist, and polymer. The most important process is to pattern a substrate by using a photolithography process that is developed previously from semiconductor industries. However, the processes need to be adjusted in order to match the dimensions and requirements for the biomedical applications.

In the literature, there are many research groups that provide solutions for micromirrors with multi-degrees-of-freedom (DOFs). While searching for better solutions, several schematic designs of actuation systems and medical micromirrors are studied for both side-imaging and forward-imaging OCT probes [7]. In general, most of the fabricated devices are based on electrostatic actuators, piezoelectric materials, bimorph materials, and electromagnetic actuators. In order to distinguish among scanning micromirrors and micropositioning devices, several methodologies are recommended. One method to distinguish these devices is by the numbers of allowable motions of the micromirror and types of the motion. Since different applications require different manipulations, this classification can help users to choose a suitable approach. For general microsurgery, a suitable scanning micromirrors should consist of three crucial DOFs; that are two rotational motions around the in-plane axis and one out-of-plane translational motion. Some research groups refer this scanning micromirror type as a tip-tilt-piston mirror. The translation along in-plane motion and the in-plane rotation are not critical since these parameters will not change directions and orientation of laser sources. In order to focus on small micromirrors, this review will focus on scanning micromirrors up to ten millimeters in size in order to emphasize on enabled technology and testing results of scanning micromirrors for biomedical applications.

In this review, classifications of micro-actuators are divided into subgroups with detailed explanations for actuation principles in Section 2. Because each type of biomedical scanning micromirrors has specific microfabrication processes and different mechanism for movement, this section is categorized by the actuation principles with a theoretical explanation and experimental results. This paper also provides recent developments and key fabrication techniques for each type of micro-actuator. For general reader, this review will be a basic guideline on scanning micromirrors and their applications in medical aspects. In Section 3, discussions are given to explain overview of recent technologies, comparisons on performances between each type of micro-actuators, current challenges, and future trends on scanning micromirrors. In Section 4 and Section 5, future work and conclusion are provided for this topical review.

## 2. Actuation Principles

Many actuators are investigated for scanning micromirrors, for example, electrostatic actuators, piezoelectric actuators, electrothermal actuators, electromagnetic actuators, pneumatic actuators, and shape memory alloy. In this section, reviews of actuation principles are discussed with examples of fabricated devices and relevant work.

### 2.1. Electrostatic Actuators

**Figure 1:**
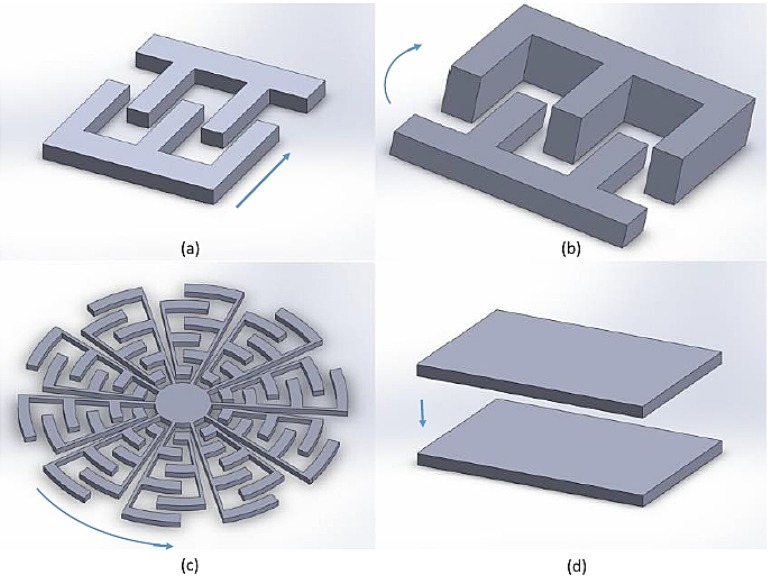
General configurations of electrostatic actuators: (**a**) linear comb-drives, (**b**) vertical comb-drives, (**c**) rotary comb-drives, and (**d**) parallel-plate actuators. An arrow indicates a motion of electrostatic actuators.

Electrostatic actuators are a device that can generate a mechanical motion by a change of stationary electric field in materials. Most of the change in electric charge occurs at the surface of the materials when there is an electrical potential between the two materials. Electrostatic actuators are implemented in many applications such as accelerometers, scanning micromirrors, photonics, televisions, and projectors. For medical application, scanning micromirrors with electrostatic actuators are investigated widely for OCT because they have fast response, large scanning angle, and low power consumption. Microfabrication processes of photolithography, thin film depositions, and high-aspect-ratio etching are usually implemented for electrostatic actuators in micromirror platforms. The common materials include silicon-on-insulator (SOI), monocrystalline silicon, polysilicon, and thin film of metals. These techniques are also implemented with MEMS processes, Multi-User MEMS Processes (MUMPs), and Complementary Metal Oxide Semiconductor (CMOS) developments with process of isotropic, anisotropic, and selective etching. This review summarizes the designs and testing for existing and potential platforms for medical applications. These electrostatic actuators can be classified into four groups; linear comb-drives, vertical comb-drives, rotary comb-drives, and surface electrostatic actuators as shown in Figure 1. Each category has a certain characteristic as explained in the following.

#### 2.1.1. Linear Comb Actuators

Linear comb actuator is a simple electrostatic comb configuration where the actuating direction is the same with the length of comb fingers. The governing equations of motion for general comb-drive actuators involved with parameters on gap between electrodes (*d*), thickness of comb finger (*t*), supplied voltage (*V*), and spring constant for beam suspensions ($k_{eff}$). The deflection ($\delta_{comb}$) of comb-drive actuators connected to beam suspensions is depicted with Equation (1).

(1) $$\delta_{comb}=\frac{F_{comb}}{k_{eff}}=n\cdot \frac{\varepsilon \cdot t\cdot V^{2}}{d}\cdot \frac{L^{3}}{4\cdot E_{spring}\cdot h\cdot b^{3}}$$

where $F_{comb}$ is the electrostatic force due to comb actuator, *n* is the number of pairs of comb fingers, *ε* is permittivity of the media, *L* is the length of the beam suspension, *E* is the Young’s modulus of the material, *b* is the width of the spring, and *h* is the height of the spring. It is also noted that the height of the device is not influenced on the deflection of linear comb-drives if the thickness of the beam suspension and comb fingers are the same.

Linear electrostatic comb-drives are implemented for several scanning micromirrors with in-plane motions [8,9,10,11,12]. The range of motion is up to 30 μm for two-axis stages. In general, high-aspect-ratio etching such as deep-reactive-ion-etching (DRIE) methods on SOI wafers, is a key for fabrication for these electrostatic actuators. These methods can be used for both creating the structures of linear comb-drives and releasing them from the substrates. Another approach is to implement a compliant actuation with high suspension stiffness for six-DOFs precision manipulator [13]. This device is designed with the micro-assembly of electrostatic actuators with submicron platform. However, the maximum strokes of these actuators are only 20 μm displacement that results to ±10 μm displacement at the end-effector. Another method to combine linear electrostatic actuators with parallel-plate actuators for 3-axis nanopositioning MEMS stage was reported by Liu *et al.* [14]. By using four sets of comb-drive for in-plane motion and parallel-electrodes for out-of-plane motion, the device was reported for a displacement of ±12.5 μm in the *X* and *Y* directions at 30 V and ±3.5 μm in the *Z* direction at 14.8 V. Moreover, the design of linear comb actuator can be implemented for angular motions of scanning micromirrors. Example of rotational stage is with torsional suspensions on two different layers of SOI substrates that can generate the lateral force for a micromirror [15]. The range of optical deflection angle is ±20.8° for one axis rotation stages. Tung *et al.* also reported a micro scanner with polydimethylsiloxane (PDMS) flexure joints for a motion range of 0.6° and 5 μm piston motion at 40 V by using linear electrostatic comb actuators [16,17]. Because of high resolution measurements and precision of linear electrostatic comb actuators, they are implemented for other sensor applications as well. Examples are a force sensor to characterize fruit fly flight behavior by using a linear electrostatic comb-drives [18] and nano newtons force-controlled manipulation of biological cells using monolithic MEMS microgripper with two-axis force feedback combs [19]. The device was reported for a force resolution of 38.5 nN and 19.9 nN for contact detection and for gripping, respectively. Additionally, Li *et al.* presented an electrostatic actuator for optical switch with a 40-μm-displacement of the mirror in VOA applications as well [20].

#### 2.1.2. Vertical Comb Actuators

Vertical comb actuators are chosen for both angular stages and transverse-displacement micromirrors. The main characteristic of vertical comb actuator is a direction of the electrostatic force that is perpendicular to the length of comb fingers. This features make it suitable for rotational scanning micromirrors. Many successful devices are demonstrated for vertical electrostatic comb-drives for scanning micromirrors in medical applications. For example, Tien *et al.* reported an implementation of vertical electrostatic comb-drive for a two-axis MEMS scanning micromirror that can produce up to 30° angles in both axes at frequency greater than 3 kHz [21,22]. With this design, the 3D endoscopic OCT was shown for bioimaging of rabbit trachea, hamster cheek pouch, and cancerous hamster cheek. Further investigations with the same design were also used for *in-vivo* images of human finger and human vocal cord [23]. With a development of angular vertical comb actuators, group of researchers led by Agguirre *et al.* demonstrated another design for electrostatic actuators with gimbal structures for 3D *in-vivo* human skin, lime pulp, and hamster cheek pouch. This device is fabricated using a foundry surface-micromachining, MUMPS, and DRIE processes. With a total dimension of 3 mm × 3 mm, the maximum static mechanical angle is ± 6° in two axes at 160 V. The resonant frequencies of the device are 140 Hz and 463 Hz [24]. Another approach for fabricating vertical comb-drives in scanning micromirrors is to bond a silicon wafer to a SOI wafer [25]. This process was shown by Kumar *et al.* for a staggered vertical comb-drives for a two-axis scanning micromirror. The device is capable for an optical deflection of ±9° at 110 V with the first resonant frequency of 385 Hz. The validations of this device for OCT imaging were reported for 3D OCT images of human skin and 2D *in-vitro* biological samples.

There are several potential designs and approaches for a scanning micromirror in medical applications, although they are not tested in clinical trials yet. The simple design is to build vertical electrostatic actuators for angular stages. For one-rotational stages, the maximum angular rotation is 46° with a design of silicon dioxide film, single-crystal silicon, and 1 μm-thick-torsion springs [26]. It is observed that most of the vertical comb-drives were fabricated on SOI wafers with a DRIE processes [27,28,29], and the combined process of DRIE and tetramethylammonium hydroxide (TMAH) [30]. For instance, Hsu *et al.* reported a flat scanning micromirror fabricated by the combined processes. The devices has a mechanical scan angle of ±10° at 30.8 kHz. Additionally, Molded Surface-micromachining and Bulk Etch Release (MOSBE) process is also investigated for vertical comb-drives with three different heights [31]. This device was reported by Wu *et al.* for an angular displacement of 1.5° at 35 V. The combined biasing schemes of AC and DC voltages are also crucial for operating vertical comb fingers [32]. Lee *et al.* demonstrated that vertical comb-drives can achieve an optical deflection angle up to 12° with a combination of 28 ${\text{V}}_{ac}$ and 35 ${\text{V}}_{dc}$.

For two-rotational micromirrors with vertical comb actuators, Zhou *et al.* implemented T-shaped torsional beam and off-axis pushing arms with the performances of 15.9° on the inner axis and 13.2° on the outer axis at 71 V [33]. Another two-DOF-rotational micromirror with vertical comb-drives was reported by Piyawattanametha *et al.* by using MUMPS technology and DRIE processes. The device was demonstrated for bi-directional operations of ±6.2° at 55 V and ±4.1° at 50 V, for the inner and outer gimbals respectively [34,35]. With a fabrication of epitaxial silicon, two-axis MEMS scanning micromirrors can achieve up to ±30.4° dynamic optical scanning angle at 40 V [36]. With V-shaped torsion hinges, the slanted vertical comb-drive can also be developed for the design of two-rotational stages. The device can achieve an optical scanning angles of 11.5° and 14° at 12 V with a resonant frequency of 247 Hz [37].

In addition, vertical comb actuators are used in translational motion for scanning micromirrors. Implementations of micromachined devices with vertical displacement are also used for an gyroscopic applications [38], and capacitive accelerometers [39]. Enabled by SOI MEMS technology, this device can obtain up to 70 μm *Z*-axis displacement at 1.5 mA. Additionally, Sandner *et al.* reported a micro scanners with electrostatic comb-drives that can generate up to ±250 μm vertical displacement [40]. This study also investigated two different suspensions of bending springs and pantograph suspension for different performances. Moreover, Wu *et al.* also reported a 2-DOF optical pick-up head with poly-silicon and silicon nitride (SiN) layers for an upward displacement of 4.6 μm at 30 V and an in-plane displacement of ±16.3 μm at 5 V [41].

For further combinations of rotational and translational stages, various designs are shown in gimbal-less monolithic silicon actuators for tip-tilt-piston. Milavonic *et al.* demonstrated up to ±30μm vertical piston by using three sets of vertical comb-drive actuators. A static optical deflection is 18° at 150 V and a resonant frequency is 4.5 kHz for both axes. Moreover, the device can be manipulated between −10° and 10° optical deflection at 4096 Hz and 1890 Hz for rotation and piston mode [42]. By implementing in-plane actuators, the scanning micromirrors with out-of-plane displacement are investigated for bi-directional tip-tilt-piston mirrors. The design with 3 layers of polysilicon process is used to fabricate a device. The performances were demonstrated for a mechanical tip and tilt angle of ±4° and a piston motion of 5 μm [43]. Another possibility is to integrate bimorph cantilever and vertical comb-drive actuators. Jeong *et al.* demonstrated this concept for a device with rotational and translational modes. This device can achieve up to 30 μm vertical amplitude at 3.5 kHz and 6.5° on 1 axis-rotation at 830 Hz [44]. Additionally, the 3-DOF stage with a vertical displacement and rotational stage can be up to 62 μm dynamically vertical displacement and ±4.7° angular displacement on both axes with 18 V [45]. This design of micromirror is enable by curled-hinge comb-drives, folded torsional springs, and CMOS structures.

#### 2.1.3. Rotary Comb Actuators

MEMS rotary comb actuator is similar to the linear comb actuator, but the configuration of the comb fingers are located along with the radius of the circular ($r_{i}$) of the device. The mathematical model for rotary comb actuators can be modified as the following equation.

(2) $$\delta_{comb}=\frac{F_{comb}}{k_{eff}}=\varepsilon \cdot t\cdot V^{2}\cdot \sum r_{i}/d\cdot k_{eff}$$

Examples of rotary comb actuators is demonstrated by Grade *et al.* for a micromirror on laser-sources tuning devices. This micromirror can scan up to a motion range of 5° [46] by using a two-beam level mechanism with rotary comb-drive actuators. However, this mirror platform is too small for laser spotsize and not suitable for OCT. Zhang *et al.* reported a rotary comb actuator with one set of comb fingers. With a separation of 2.5 μm between 2-μm-width comb fingers, the device is capable for a rotation angle of 4.7° [47]. It is observed that the flexure hinges are mostly implemented for rotary comb actuators, instead of spring suspension. This design distinguishes rotary comb-drives from other type of electrostatic actuators. Moreover, Yeh *et al.* reported a full rotary comb actuators based on SOI substrate [48,49]. This full rotary comb actuators can generate a rotational angle of 2.6° at 5 V. For other applications, rotary comb actuators are also investigated for MEMS energy-harvesting device. A rotary comb with 6-mm-diameter, 30 μm thick springs, and 3.6 μm comb gap was designed [50]. In MEMS variable optical attenuator, rotary comb actuators with 2.4-mm-diameter, and 80 μm structural thickness was used [51]. The maximum rotation angle of this device is 2.4°. For the applications of OCT, the rotary comb actuators was proposed by Ayers *et al.* [52]. This device implements photoresist hinges to assembly a scanning micromirror. However, the validification of this design for OCT are not shown in public yet.

#### 2.1.4. Parallel-Plate Actuators

Parallel-plate electrostatic actuators are alternative for micromirrors in various applications. By using larger surface areas, the designs of scanning micromirror can be developed. Theoretically, electrostatic force of surface electrostatic actuators can be formulated in the following equation.

(3) $$F_{surface}=\varepsilon \cdot V^{2}\cdot l_{surf}\cdot w_{surf}/2\cdot d^{2}$$

where $l_{surf}$ and $w_{surf}$ are the length and width of surface electrodes. The rests of the parameters are still the same with Equation (1) for linear comb-drives. Example of micromirror with surface electrostatic actuators for 3-D OCT tested in biological samples is demonstrated by Yeow *et al.* [53]. The device contains a 1.1-μm-thick SiN hinges and surface electrodes. With a platform’s dimension of 1.4 mm × 1.7 mm, the performances of the device are at 0.3° on two axes at 55 V with a resonant frequency of 181 Hz and 45 Hz for outer frame and mirror respectively. Moreover, surface electrostatic actuators can perform up to three DOFs by using a CMOS technique [54]. For a tip-tilt-piston stage, Kao *et al.* reported an electrostatic phenomenon of parallel plates. This motion can be implemented for micro-manipulation up to 2.1 μm piston stroke and 2.55° tilting angle at 40 V. Example of surface electrostatic actuators for out-of-plane translation are used in many designs [55,56,57,58,59]. For example, micromirrors with surface electrodes can be used to generate up to a 1.2 μm-vertical displacement at 60 V [57]. Pan *et al.* also reported micromirrors with surface electrostatic actuators with a maximum piston motion of 50 μm at 100 V [55]. Another design by He *et al.* also demonstrated a repulsive-force for out-of-plane motion with the interdigitated comb configurations [58]. Fabricated by PolyMUMPs technology, this device can achieve a static motion of 86 μm at 200 V and the mechanical rotation range of ±1.5° in two axes.

Surface electrodes are also used for rotational stages for both one DOF and two DOFs. For one-axis micromirrors, the range of motion can be up to 9° for SOI microfabrication [60,61,62,63]. Bulk silicon materials [64,65,66,67] and monocrystalline silicon [68] are also validated for this methodology as well. Examples of one-rotational stages were shown by Hao *et al.* for a micromirror with a static rotation angle of 3° on both axes at 40 V with the first resonant frequency of 1100 Hz [69]. For two-axis rotation stages, the scanning micromirror can be fabricated on a single-crystalline silicon for an optical scan angle of ±7.5° [70]. Another two-axis mirrors can be fabricated on SOI wafers with a mirror dimension of 750 μm × 800 μm. This device has a mechanically stable operation of ±5° with 60 V [71]. Crystalline silicon with alignment and bonding technique is another technique for a fringe-field tilting mirror with 8° scanning range at 142 V [63]. Additionally, two-axis rotational stage can be implemented with a sidewall electrodes. This fabricated device had a mechanically rotation angle of ±11° in a static mode [72]. Moreover, Zara *et al.* investigated an integrated force array (IFA) method for OCT scanning in medical applications as well. By using capacitive cells contraction due to electrostatic force, polyimide conductive strips can generate a motion up to 77° and 142° at a resonance of 20.6 Hz and 41.2 Hz and 50° static. The samples of *in-vitro* porcine colon and eyeball are reported for this device [73,74,75]. Because of the larger area for electrostatic charges, the induced force can be higher. However, the gap distance is also crucial for the design. If the area is larger, the gap distance is always larger and this will reduce the electrostatic force.

**Figure 2:**
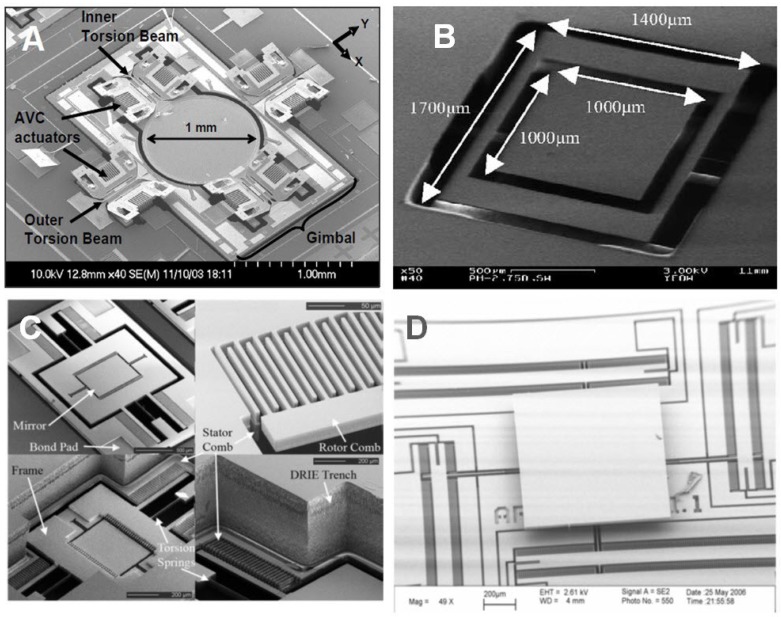
Several designs of scanning micromirrors with electrostatic actuators: (**A**) two-axis microelectromechanical systems (MEMS) scanning catheter with vertical comb-drive by Aguirre *et al.* [24], (**B**) micromachined 2-D scanner with surface electrostatic actuators by Yeow *et al.* [53], (**C**) two-axis MEMS scanning micromirror with staggered vertical comb-drive by Kumar *et al.* [25], and (**D**) two-axis MEMS scanning micromirror with vertical comb-drives [23]. Reproduced with permission from [23,24,25,53].

To summarize the performances of scanning micromirrors by using electrostatic actuators, Table 1 compares all references from literature reviews based on the subgroup of electrostatic actuators. Moreover, some samples of scanning micromirrors by vertical comb-drives are shown in Figure 2. In addition, Figure 2d shows example of micromirror with surface electrostatic actuators for 3-D OCT tested in biological samples is demonstrated by Yeow *et al.* [53].

**Table 1:** Relevant work on micromirrors with electrostatic actuators.

Reference	Year	Size (in mm)	Characteristics	Operating Conditions	Natural Frequency
***Linear Comb Actuators***					
Milanovic *et al.* [15]	2001	0.7 mirror	±20.8° optical (2 axis)	90 V	2 kHz
Sun *et al.* [11]	2002	3.2 × 3.0	4.5 μm, 1.5 μm	10 ${\text{V}}_{x}$, 68 ${\text{V}}_{y}$	-
Li *et al.* [20]	2003	-	45 μm	35 V	-
Tung *et al.* [16]	2005	2.5 × 2.5	±0.6°, 5 μm	40 V	5 kHz
Liu *et al.* [14]	2007	4 × 4	12.5, 12.5, 3.5 μm	30 V	-
Mukhopadhyay *et al.* [9]	2008	-	1.72°, 18 μm, 18 μm	85 V	465 Hz
Kim *et al.* [19]	2008	-	57 μm	9 V	-
Laszczyk *et al.* [8]	2010	10 × 10	30 μm	100 V	290 and 550 Hz
Brouwer *et al.* [13]	2010	4.9 × 5.2 mm	20, 20, 20 μm	105 V	3800 Hz
He *et al.* [58]	2011	3.2 × 3.2	±1.5°, 86 μm	200 V	1 kHz
Chu *et al.* [37]	2011	8 × 8	14°, 11.5°	12 V	247 Hz
***Vertical Comb Actuators***					
Conant *et al.* [27]	2000	0.55 dia mirror	24.9°	250 V	34 kHz
Patterson *et al.* [29]	2002	1 × 1 mirror	18°	110 V	1400 Hz
Xie *et al.* [38]	2002	0.6 × 0.6	270 μm	14 V	5.08 kHz
Lee *et al.* [32]	2002	1.5 × 1.2 mirror	12°	28 ${\text{V}}_{ac}$, 35 ${\text{V}}_{dc}$	1353 Hz
Xie *et al.* [45]	2003	1 × 1 mirror	4.7°	25 V	233 Hz
Milanovic *et al.* [42]	2004	0.4 × 0.4	10°, 10°, 30 μm	150 V	1890 Hz
Lee [36]	2004	1.5 × 1.5	±15.2° mech	40 V	1340 Hz
Jeong *et al.* [44]	2005	0.3 dia	6.5°, 3 μm	5.5 V	830 Hz
Jung *et al.* [21]	2005	2.5 × 3 die	30° both axis	100 V	8 kHz
Piyawattanametha *et al.* [34]	2005	-	12.4° and 8.2°	55 V	144 Hz
Chong *et al.* [28]	2006	-	8°	5 V	350 Hz
Jung *et al.* [22]	2006	1.2 mm mirror	20° optical	100 V	2.4 kHz
Wu *et al.* [31]	2006	0.8 × 0.8 mirror	1.5° (2-axis)	35 V	3.8 kHz
Wu *et al.* [31]	2006	-	16.3, 16.3, 4.1 μm	30 V	1900 Hz
Zhou *et al.* [33]	2006	0.8 × 0.8 mirror	21.8° one axis	75 V	3.6 kHz
Aguirre *et al.* [24]	2007	3 × 3	±6° mech. (2-axis)	160 V	140 and 463 Hz
Pardo *et al.* [43]	2007	0.12 × 0.12 mirror	8°, 5 μm	110 V	-
Wu *et al.* [26]	2007	0.2 × 0.15	46°	140 V	-
Hsu *et al.* [30]	2008	1 mm dia	10° optical	100 V	30 kHz
Kumar *et al.* [25]	2008	2 × 2.5 die	±9° (2 axis)	110 V	385 Hz
Sandner *et al.* [40]	2009	3 dia mirror	100 μm	44 V	500 Hz
		1.1 × 1.5	250 μm	30 V	5000 Hz
***Rotary Comb Actuators***					
Ayers *et al.* [52]	2004	<1 mm diameter	-	-	-
Grade *et al.* [46]	2004	4.3 × 3 chip	5°, 300 μm	150 V	300 Hz
Yeh *et al.* [48]	2006	3.2 × 4.7 chip	2.6°	5 V	400 Hz
Zhang *et al.* [47]	2007	1.5 mm long	4.7°	70 V	-
***Surface Electrostatic Actuators***					
Su *et al.* [70]	2001	0.48 × 0.46 mirror	7.5°	-	-
Zara *et al.* [73,74,75]	2002	IFA method	77° and 142°	65 V	-
	2002	(1.5 mirror)	50° optical, 146°	50 V	20.6 Hz
	2003	2 × 2.25 mm	-	-	-
Niklaus *et al.* [68]	2003	16 μm × 16 μm	0.8 μm gap (one axis)	12.5 V	-
Greywall *et al.* [63]	2003	-	8°	142 V	-
Greywall *et al.* [63]	2003	-	9°	210 V	-
Dokmeci *et al.* [71]	2004	0.75 × 0.8 mirror	5°	60 V	175 Hz
Pan [55]	2004	-	50 μm	100 V	-
Yeow *et al.* [53]	2005	1.4 × 1.7	-	100V	45 and 181 Hz
Kudrle *et al.* [61]	2005	70 × 70 for 1296 mirrors	5° mech.	160 V	78 and 187 Hz
Yan *et al.* [60]	2005	0.2 × 0.2 mirror	0.5°	2.7 V	50 Hz
Cheng *et al.* [66]	2005	-	5 μm	22.5 V	-
Kallweit *et al.* [64]	2006	0.5 × 0.5	2.5° (one axis)	300 V	-
Singh *et al.* [65]	2006	10 × 10	2°	50 V	-
Joudrey *et al.* [67]	2006	8 × 3	2°	200 V	1000 Hz
Ya’akobovitz *et al.* [62]	2008	2 × 2 plate	10° (one axis)	20 V	3.8 kHz
Kao *et al.* [54]	2009	50 μm mirror	2.25° both axis	40 V	59.1 kHz
			(Displacement up 2.10 μm)		
Hu *et al.* [57,59]	2010	0.4 × 0.4 mirror	1.8°, 1.65 μm	100 V	2.5 kHz
Zhang *et al.* [12]	2010	-	5°	30 V	-
Bai *et al.* [72]	2010	1 × 1 mirror	11°	55 V, 240 V	-
Michael *et al.* [56]	2012	0.2 × 0.8 mirror	27 μm	17 V	-

### 2.2. Piezoelectric Actuators

Piezoelectric actuators are governed by the principle that stress in the material are caused by a change in the electrical field applied to the actuators. Two different material with different piezoelectric properties can be extended or contracted when they are subjected to an electric field. This type of the piezoelectric actuator is known as a bimorph actuator. The other example is when only one material is piezoelectric material, and the other material is not active. This latter type of piezoelectric actuator is also known as a unimorph actuator. Although scanning micromirrors with piezoelectric actuators are not investigated as much as the other types, there are possible solutions for fabricating a scanning micromirror with piezoelectric actuators [76,77]. The advantages of piezoelectric actuators are fast response, low driving voltage, and low power consumption. The common characteristic equation of the piezoelectric actuators are related to the strain mismatch between piezoelectric materials and it can be formulated as shown in Equation (2).

(4) $$\delta_{piezo}=\frac{C\cdot L^{2}}{t_{p}\cdot \left(t_{p}+t_{x}\right)}\cdot d_{31}\cdot V$$

where $\delta_{piezo}$ is the deflection of piezoelectric layer, *d*_31_ is a piezoelectric charge constant or a polarization generated per unit of mechanical stress applied to a piezoelectric material. *C* is a constant for each material and *V* is an applied voltage. *L*, $t_{p}$, and $t_{x}$ are length of piezoelectric beam, thickness of piezoelectric layer, and thickness of supporting layer, respectively.

To classify the study on scanning micromirrors with piezoelectric actuators, two groups of researches are distinguished by the processes of formation. One is the scanning micromirrors made from bulk lead zirconate titanate (PZT) material with the final thickness about 20–40 μm. The other is the micromirrors made from thin film PZT with the final thickness in the range of 1–5 μm. Both of them are prepared by different processes in microfabrication techniques and have distinguish characteristics that can be explained in the following.

#### 2.2.1. Bulk Lead Zirconate Titanate (PZT)

The general form of piezoelectric actuator is bulk PZT that can be polished for a thinner component of scanning micromirrors. In general, the thinner PZT can deflect more than the thicker PZT as shown in Equation (2). The typical micromirror platform can be fabricated with this type of piezoelectric materials by the process of chemical or mechanical polishing process. Then, the piezoelectric material can be patterned or etched by combinations of various solutions such as HCl, HNO_3_, HF, BHF, KOH, NaOH, and NH_4_Cl. These wet etching processes are fast, easy to implement, and low cost. However, the undercut issues, sidewall profiles, etch rate control, and material selectivity are important challenges for these fabrication processes. Example of a tip-tilt-piston micropositioning stage with the lapping process of bulk PZT substrate was demonstrated by Aktaka *et al.* [78]. The final thickness of PZT layer is 17 μm and the stage size is 3 mm × 3 mm. The static motion of the device is ±21 μm and ±1.15° under 25 V driving voltage. The first resonant frequency of the device is 0.9 kHz. The maximum power consumption is 450 μW. The design of six-DOF biomedical mirror is also developed of the same design. The device has a maximum static displacement of ±1° for rotation mode, ±7.5 μm for *X*/*Y*-displacement mode, and ±22 μm for *Z*-displacement mode [79]. Moreover, Wilson *et al.* also reported the mechanical thinning of PZT ceramics with bonding layer of printed circuit board (PCB) adhesive [80]. With the final thickness of 40 μm and 4 mm long, this PZT cantilever can generate ±70 μm displacement. Additionally, Xu *et al.* described a thinning process for bulk PZT by using wet-etching method in BHF/HCl/NH_4_Cl solution. In this design, the final thickness of the PZT layer is 40 μm. After the fabrication of the device, arrays of actuators can obtain the maximum deflection of 4.5 μm at 100 V and 21 kHz resonant frequency [81]. With a cantilever of 10 mm-length and 5 μm-thick PZT-Au-Si actuator, the device can deflect up to 200 μm at 100 V and 815 Hz resonant frequency [82]. Even though the thinner thickness of the bulk PZT can be lapped, another crucial issue for bulk PZT fabrication is bonding processes between bulk PZT and base materials. Epoxy materials, such as, solder materials, gold intermediate layer, resin, and benzocyclobutene (BCB) adhesive, and silver paste are developed to glue the base materials to bulk PZT for these processes. Still, the fabrication and integration processes are complicated for scanning micromirrors with higher DOFs.

#### 2.2.2. PZT Thin Film

In order to improve on the performances of the scanning micromirror, PZT thin film are usually implemented using several methods. To deposit PZT thin film, processes in MEMS/CMOS are developed such as arc discharged reactive ion-plating (ADRIP) [83], epitaxial process [84], sol-gel spin-coating [85,86], and sputtering [87]. The thickness of the PZT thin film is generally around 0.4–3 μm. After the deposition, both wet and dry etching processes can be performed to pattern these thin film piezoelectric materials. The wet etching chemicals and processes are similar to those of bulk PZT. In addition, the dry processes can be used for thin film PZT with a combinations of SF_6_ and CF_4_ gas with argon gas. These dry process has advantages for low undercut and high resolution, though the selectivity of mask is still challenging for the development of PZT thin film process.

For beam configurations of thin film PZT, sol-gel piezoelectric is common material that are used. Several cantilever designs are implemented for two-dimensional (2D) scanning micromirrors. By using a bending and torsion motion for 3 mm × 3 mm micromirror, Koh *et al.* reported for sol-gel PZT techniques [88]. The thickness of this PZT beam is 3 μm, and 500 μm in length. Biasing schemes are altered for 10 PZT stripes to generate motion for this design. The first resonant frequency is at 122 Hz and 2.46 kHz for bending and torsional mode. The maximum optical deflection angles are 1.15° and 0.2° for bending and torsional mode at 1.5 V. For a larger micromirror (5 mm × 5 mm), the device can generate higher maximum deflection angles of ±8° at 9 V in bending mode and ±4.6° at 8 V in torsional mode [89]. In addition to a linear cantilever configuration, 2D-scanning micromirrors were investigated with an S-shaped cantilever. With a micromirror size of 1.65 mm × 2 mm, the maximum optical deflection angles at 3 V are ±38.9° and ±2.1° for bending and torsional modes at a resonant frequency of 27 Hz and 70 Hz respectively [90]. A static optical deflection angle of these devices is also reported for 4.6° at 10 V [91]. Moreover, Gilchrist *et al.* investigated another one rotation micromirror with a combination of thin PZT material, silicon dioxide, and SiN thin film [92]. With a size of 600 μm × 840 μm micromirror, this cantilever had a static angular displacement up to ±7° with a resonant frequencies about 600 Hz.

Moreover, thin film sol-gel piezoelectric actuators were investigated for a 2D micromirror in several configurations. With the PZT thickness of 0.7 μm, the micromirror is connect to with four actuators in the work of Tsaur *et al.* [93]. The testing results showed a scanning angle range up to 26° with 7.5 V at 3750 Hz. To avoid the deformation of the scanner, two layers of PZT with a thickness of 700 nm can be deposited on both sides of the Pt/Ti layer. Moreover, Smite *et al.* reported that the design with two actuators can achieve a maximum deflection of 180 μm at 18 V. A static optical angle of this micromirror is up to 40° at 13 V [94]. Additionally, PZT unimorph with a gimbal and flexure hinges are also used for micromirror arrays [95]. This device can move up to ±0.75° at 15 V for *X* and *Y* rotation. Further developments by Qui *et al.* also showed the vertical translational actuators by using thin film PZT as well [96]. By using four of 920 μm × 70 μm beams, this prototype has a performances up to 120 μm static displacement. Nevertheless, PZT material can be implemented in other different ways, for example, H-shaped cavity with three-wafer stack bonding [97], and piezoelectric fiber actuator [98]. Though these methods and technologies are in progress, piezoelectric materials are potential candidates for scanning micromirrors for medical applications with further developments.

Example of a tip-tilt-piston (3-DOFs) micromirror was demonstrated by Zhu *et al.* for a micromirror based on sol-gel PZT with a thickness of 0.6 μm and silicon dioxide with a thickness of 1 μm [76]. The device is with four piezoelectric unimorph actuators connected to a rectangle micromirror. Each set of lateral shift design consists of three piezoelectric beams made of thin film Pt/Ti/PZT/Pt/Ti/SiO_2_. The dimension of the scanning micromirror is 1.1 mm × 1.1 mm and a chip footprint is 2 mm × 2 mm. The resonant frequency of the device is at 316 Hz and 582 Hz for the piston and rotation modes. The maximum piston motion at the resonant frequency is about 32 μm and two-dimensional rotating scan ranges are 5° at 2 V. Liu *et al.* also reported a similar approach with double-S-shaped piezoelectric actuators [77]. The resonant frequency of the rotation modes is at 3.5 kHz. The static motion of the device is 27 μm and ±2° under 5 V driving voltage. The dynamic motion of the device is 109 μm and ±9.65° under 2 V sine wave resonant driving voltage. While the thickness of the piezoelectric is important for the scanning micromirrors, the strength of material and ultimate stress need to be considered for the design as well.

**Figure 3:**
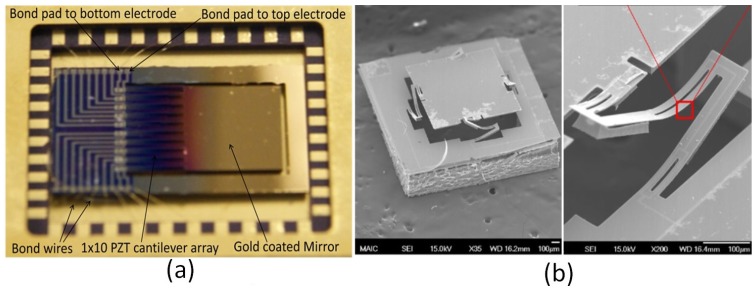
Several designs of scanning micromirrors with piezoelectric actuators: (**a**) a piezoelectric actuator in cantilever configuration by Koh *et al.* [89], and (**b**) a piezoelectric actuator in parallel kinematics [76]. Reproduced with permission from [76,89].

Recent developments of scanning micromirror with piezoelectric actuators are compared in Table 2. Moreover, some examples of scanning micromirrors with piezoelectric actuators are shown in Figure 3.

**Table 2:** Relevant work on micromirrors with piezoelectric actuators.

Reference	Year	Size (in mm)	Characteristics	Operating Conditions	Natural Frequency
Yee *et al.* [95]	2001	2 × 2	±0.75° (2D)	±15V	-
Tsaur *et al.* [93]	2002	3 × 3	26°/24° (2D)	15 V	3750 Hz
Smits *et al.* [94]	2005	1.3 × 1.3 mirror	40°, 180 μm	18 V	17.4 kHz
Gao *et al.* [97]	2006	1 × 1.25	0.0123 μm	7.5 V	1.5 kHz
Kim *et al.* [85]	2008	-	5.5°(*X*), 4.7°(*Y*)	16 V	1.7 kHz
Gilchrist *et al.* [92]	2009	0.6 × 0.84	±7°	10 V	1–2 kHz
Koh *et al.* [88]	2010	3 × 3 mirror	2° DC/ 5° AC, 35 μm	3 V	122 Hz
Qiu *et al.* [96]	2010	1.7 × 1.7	90 μm	20V	240 Hz
Zhu *et al.* [76]	2011	2 × 2	5° (2D), 32 μm	2 V	316 Hz
Koh *et al.* [89]	2011	5 × 5 mirror	±8° bending and ±4.6° torsion	9 V	30 Hz
Koh *et al.* [90]	2011	4.2 × 5.2	±38° bending and ±2° torsion	3 V	27 Hz
Pan *et al.* [98]	2011	1 × 1 mirror with fiber	17.9°/2.6°	400 V	6780 Hz
Koh *et al.* [91]	2012	-	4.6°	10 V	27 Hz
Liu *et al.* [77]	2013	2 × 2	±2°, 27 μm	5 V	2.4 kHz
Aktakka *et al.* [78]	2013	3 × 3 (2.3 mirror)	±1.15° (3D), ±21 μm	25 V	930 Hz

### 2.3. Electrothermal Actuators

Electrothermal actuator is a micromachined device that can generate a motion by an expansion of materials due to different thermal expansion coefficients of two materials. In general, the change in piezoelectric and thermal property of materials can cause the motion at the same time for bimorph or unimorph material. Theoretically, the deflection of electrothermal cantilevers (Δ*L*) can be formulated as a function of length of actuators (*L*), difference on thermal expansion coefficient of two materials (Δ*α*), and temperature difference during operation (Δ*T*). The equation of motion can be written as shown the following equation.

(5) ΔL=L·Δα·ΔT

Examples of electrothermal actuators for medical applications include scanning micromirrors, endoscopy, and OCT. The microfabrication processes of electrothermal actuators involves common thin film materials of aluminum, silicon dioxide, polysilicon, and heating metals (such as platinum or tungsten). The classifications of electrothermal actuators can be distinguished into two group by the shapes and its configuration as shown in the following.

#### 2.3.1. Cantilever Micromirror

Various shapes of actuators are investigated for electrothermal actuators. For example, Henneken *et al.* reported the U- and V-beam thermal actuators [99] with a deflection up to 14 μm. Schweizer *et al.* demonstrated a two-dimensional micromirror with "L"-shaped cantilever and Physical Vapour Deposition (PVD) hinge [100] for a mechanical scanning range of 15° in two directions. With PVD metal layer, bimorph beam can achieve an out-of-plane motion up to 90° for mechanical scan angles with resonant frequencies between 100 Hz and 600 Hz [101]. Nickel Z-shaped beam was also used for 2-DOF MEMS nanopositioner with eletrothermal actuation [102]. Moreover, Liu *et al.* reported an electrothermal actuator with a curved concentric connection. The micromirror is connected to four legs of actuators and each leg consists of three sets of bimorph beam. The performances of the device is 11° and 200 μm [103].

Many research groups have also implemented cantilever actuation with several designs and materials. In the early development of bi-axial scanning micromirror, aluminum and silicon dioxide thin film are investigated by Buser *et al.* The device can provide a deflection angle of 8° at 180 mW with a cantilever structure [104]. In 1995, Buhler demonstrated a bimorph micromirror with aluminum, silicon dioxide, and polysilicon heating. The device is fabricated by ethylenediamine-pyrocatechol (EDP) anisotropic etching. For a size of 40 μm cantilever, the finite element analysis showed a maximum deflection of 14 μm and a rotation angle of 4.6° with 4.6 mW heating power [105]. In 2001, Pan *et al.* reported array of bimorph of aluminum and silicon dioxide for OCT and two dimensional endoscopy of *in vivo* porcine bladder through cystectomy [106]. In addition to aluminum and silicon dioxide, SiC cantilever with platinum and NiCr electrodes was reported for electrothermal actuators by Jiang *et al.* [107]. The resonant frequency of the device is 117 kHz. With a technique of MUMPs process with polysilicon cantilever, buckle-beam structure can obtain a static deflection of 18° at 8 kHz and 160 mW power consumption [108]. Moreover, a semicircular multimorph layer with aluminum-tungsten can be used for electrothermal actuator with low driving voltage at 0.68 V. The allowable scanning angle of the device is 60° at 11 mW power input [109].

Furthermore, a collaborated group of researchers in Singapore investigated a scanning micromirror by implementing electrothermal cantilever combined of silicon, silicon dioxide, and aluminum heater [110]. For a 1.5-μm-thick SOI substrate, a maximum tilting angle is 17° at 1.5 V. The chip size is 1.5 mm × 1.5 mm for a micromirror plate size of 400 μm in diameter. The packaging technique of silicon optical bench (SiOB) was used to assembly this micromirror with a Gradient-index (GRIN) lens for a 4 mm polycarbonate tube [111]. OCT testings of this device can be used to construct 3D images for *in vivo* and *en face* diagnostics [112]. Several OCT bioimaging technologies were tested for *in-vitro* onion [113,114], *ex vivo* mouse muscle, and mouse skin [115].

#### 2.3.2. Parallel-Connected Micromirror

Another interesting research in electrothermal actuators for endoscopic devices are studied by Professor Huikai Xie. In this design, micromirror cantilevers with aluminum and silicon dioxide mesh were fabricated on a single-crystalline silicon by a DRIE process. The micromirror was reported for 17° at 15 mA. The device has a resonant frequency of 165 Hz with an operating current of 12 mA [116]. *In vivo* 2D images of porcine bladder [38] and *ex-vivo* images of rabbit bladder [117] were tested for bioimaging of this device. In 2003, Xie *et al.* reported an improvement on performances of similar structures with arrays of bimorph actuators for an optical scanning angle of 35°. More images are demonstrated for *in vivo* diagnosis of rat bladder cancers as well [118]. In similar design, Jain *et al.* developed a two-axis micromirror with similar structure [119]. The device consists of an orthogonal set of bimorph beam embedded inside the movable frame. The maximum rotation angle of the micromirror and frame is 64° and 33°, respectively. Additionally, miniature endoscopic OCT probe with two axes scanning micromirror can be implemented with three sets of folded bimorph actuators in series [120]. Four sets of these serial bimorph are connected to each sides of a rectangle stage. Bimorph film with Al/SiO_2_ is used for actuating for a range of ±16° at 3.6 V. The first resonant frequency of the device is 659 Hz. The device has 1.5 mm footprint and 2.6 mm probe diameter. The testings of the device are used for recording images of microspheres in PDMS and rat brain tissue. In 2010, Sun *et al.* reported further developments of these techniques. The electrothermal actuators consist of 4 legs of rectangle bimorph and 3 arrays for each set [121]. This device has a piston motion of 600 μm at 5.5 V and ±30° motion around both axes for a micromirror of 1 mm × 1 mm. The total footprint of the MEMS micromirror is 2 mm × 2 mm. The device is tested with a Lissajous scan pattern and 3D *in vivo* images of mouse tongue and ear. Moreover, a piston motion of the micromirror was demonstrated by Izhar *et al.* by using aluminum, polysilicon electrothermal actuators with embedded heaters, and polysilicon flexural connectors [122]. The device can reach a maximum vertical displacements of 131 μm and rotating angles of 32°. The cut-off frequency of the device is 10.5 Hz with the power consumption of 12 mW.

For scanning micromirrors with translation and rotation, The devices with two sets of bimorph can generate a vertical displacement of 200 μm and a rotation angle of ±15° at 6 V with a size of 0.7 mm × 0.32 mm micromirror [123]. A tip-tilt-piston stage was also demonstrated for a motion of 480 μm in *Z*-axis and ±30° about *X* and *Y* axis for a voltage less than 8 V by using bimorph of aluminum and silicon dioxide (Al/SiO_2_) and platinum heater [124]. This micromirror has a dimension of 40 μm × 1000 μm × 1000 μm with the first resonant frequencies of 336 Hz. Moreover, a single-crystal silicon micromirror can be used to create sets of bimorph beam to manipulate an inner frame and micromirror. The device with four sets of bimorph were demonstrated with a range of 500 μm piston motion at 15 V and a maximum optical scan angle of 7°. For one degree of rotation, the maximum optical scan angle of the device is 66° at 8.5 V [125]. Wu *et al.* also demonstrated a tilting angle with three sets of bimorph for a performances of 0.7° tilting angle and 620 μm vertical displacement at 5.3 V [126]. Additionally, Todd *et al.* reported the use of four inverted-series-connected (ISC) bimorph in rectangle configuration. The device is fabricated by the AMI 1.5 μm CMOS process [127] and the experimental results showed a maximum displacement of 56 μm at temperature of 150°C.

**Figure 4:**
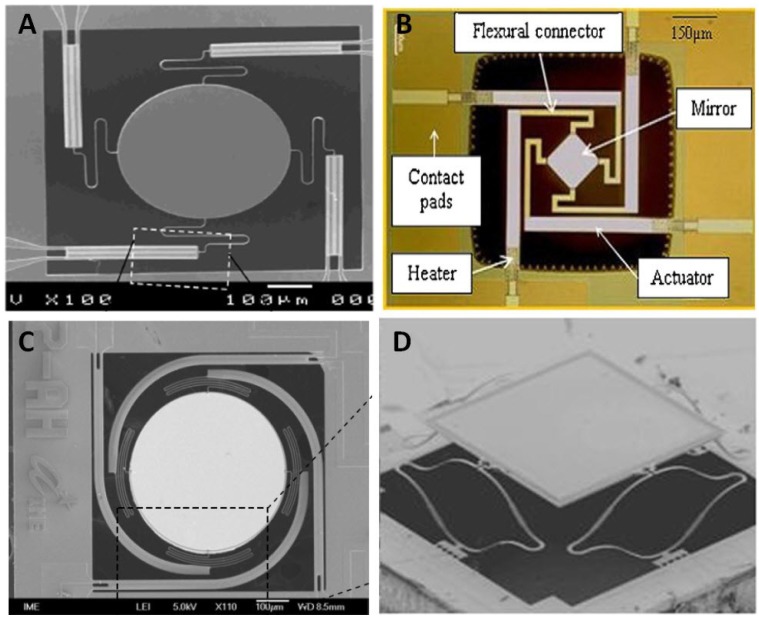
Designs of MEMS scanning micromirror with electrothermal actuators with four sets of actuators: (**A**) integrated endoscopic probe by Mu *et al.* [115], (**B**) by Lzhar *et al.* [122], (**C**) by Singh *et al.* [110], and (**D**) by Xie *et al.* [121,124]. Reproduced with permission from [110,115,121,122,124].

Relevant work on electrothermal actuators for scanning micromirrors are shown in Table 3. Some examples of scanning micromirrors in recent development are shown in Figure 4.

**Table 3:** Relevant work on micromirrors with electrothermal actuators.

Reference	Year	Size (in mm)	Characteristics	Operating Conditions	Natural Frequency
Buser *et al.* [104]	1992	-	8°	180 mW	-
Buhler *et al.* [105]	1995	0.035 × 0.04	4.5°	-	-
Schweizer *et al.* [101]	1999	-	90°	1 mW	330 Hz
Schweizer *et al.* [100]	2000	-	30°	5 mW	220 Hz
Pan *et al.* [106]	2001	-	15°	-	165 Hz
Xie *et al.* [38]	2002	1 × 1 mirror	32° optical	12 mA	-
Xie *et al.* [128]	2003	1 × 1 mirror	35° optical	7 mA	-
Xie *et al.* [116]	2003	1 × 1 mirror	32° optical	12 mA	165 Hz
Xie *et al.* [118]	2003	1 × 1 mirror	37° optical	7 mA	-
Jain *et al.* [119]	2004	1 × 1 mirror	64° and 33° (2R)	8 mA	259 Hz
Jain *et al.* [123]	2005	0.7 × 0.32 (0.19 mirror)	26.5°, ±15, 200 μm	6 V	1.18 kHz
Todd *et al.* [127]	2006	0.5 × 0.5 mirror	56 μm	-	-
Jiang *et al.* [107]	2006	50 μm cantilever	300 nm	0.2 V	117 kHz
Henneken *et al.* [99]	2006	2 mm length	13 μm	45 V	-
Jain *et al.* [125]	2006	0.5 × 0.5 mirror	±30° (2R), 500 μm	12 V	170 Hz
Singh *et al.* [110]	2008	1 × 1	17°, 250 μm	2 V	-
Xu *et al.* [112]	2008	2.5 × 2.5	17° mech.	1.5 V	46 Hz cut-off
Wu *et al.* [126]	2008	2.5 × 2.5	0.7° tilt, 620 μm	5.3 V	500 Hz
Jia *et al.* [124]	2009	1 × 1 mirror	±30° (2R), 480 μm	8 V	336 Hz
Premachandran *et al.* [111]	2009	1.5 × 1.5 chip	16°	-	-
Wang *et al.* [113]	2010	1.5 × 1.5	11°	1.2 V	60 Hz
Sun *et al.* [121]	2010	-	30° (2R), 600 μm	5.5 V	13 Hz
Mu *et al.* [115]	2011	1 mm dia. mirror	11°	1.4 V	75 Hz
Pal *et al.* [109]	2011	1 mm dia. mirror	60° (2R)	0.6 V	104 Hz
Liu *et al.* [120]	2011	1.5 × 1.5	±16° (2 layer flip)	3.6 V	659 Hz
Izhar *et al.* [122]	2011	4.5 die 1.3	32° optical, 131 μm	12 mW	10.5 Hz cut-off
Liu *et al.* [103]	2012	2 × 2	±11°, 227 μm	0.6 V	197 Hz

### 2.4. Magnetic Actuators

A change in electrical field can cause a motion in electromagnetic actuators that can be implemented for medical applications as well. Example of scanning micromirrors with magnetic actuators for OCT is demonstrated by Kim *et al.* Two-axis magnetically-driven MEMS scanning catheter for endoscopic consists of four folded flexure hinges and a manually-glued neodymium magnet (NdFeB) at the back of the micromachined micromirrors [129]. The device has a range of ±20° in optical scanning angle. The assembled catheter has an outer diameter of 2.8 mm, where contains of coil of American Wire Gauge (AWG) wire for slow and fast coil pairs, GRIN lens, and optical fiber. With this device, *in vivo* oral cavity tissues and a 3D image of *in vivo* fingertip were demonstrated. Another example of two-axis micromachined scanners is a nickel-plated magnet on a bulk stainless steel plate in a gimballed cantilever and a gimballed torsional configuration [130]. Enabled by the patterning of stainless steel plate, an electrochemical cell with HCl etching is used to etch the plate and two magnets with a size of 1 mm × 5 mm × 1.5 mm were attached into the frame. The testing results for a gimballed cantilever showed an optical scanning angle of 11.7° and 23.2° in each directional. The device with a gimballed torsional beam can achieve an optical scanning angle of 5.9° and 76° in each directional. Furthermore, electromagnetic MEMS micromirror technology for 3-D optical switching application was demonstrated by Berstein *et al.* for the mechanical rotation of 8° at 0.75 mA and the first resonant frequency of 96 Hz [131]. Fujita *et al.* also demonstrated 2-axis MEMS micromirror with SU-8 torsion beam and external samarium–cobalt (SmCo) magnet. The platform has an optical scanning angle up to ±40° for a resonant mode and 5° for a static mode [132]. Ahn *et al.* also reported a two-DOF stage with current routing by using a single permanent magnet to produce torque from the Lorentz force in the external magnetic field from a single permanent magnet under the micromirror [133]. The device is made of 20-μm-thick chemical mechanical polishing (CMP) silicon, Plasma-Enhanced Chemical Vapor Deposition (PECVD) silicon dioxide, and aluminum thin film. The micromirror dimension is 3.5 mm × 3.5 mm. The device can achieve a maximum angle of rotation of ±1.51° and ±5.71° for micromirror and movable frame with a first resonant frequency of 920 Hz. By electroplating of a copper coil, the optical scanner can be actuated with an external magnetic field as well [134]. The device can achieve ±4.35° for *X*-axis and ±15.7° for *Y*-axis at 4.2 V and 1.76 V. Other material, such as, chromium and gold can be patterned for a magnetic coil for micromirror as well. Mitsui *et al.* reported a device with these multi-layer coil, polyimide insulator, and silicon torsion beams [135]. The device consists of 4 sets and 2 sets of planar coils for *Y* axis and *X*-axis on movable plate. The maximum static optical scanning angle of the device is ±8° for a current of ±4.6 mA and ±10.3 mA with the resonant frequency of 106 Hz and 80.5 Hz in *X*- and *Y*-axes, respectively.

Although magnetic actuators are used in several medical applications, there are limitations for external magnet and integration processes with magnetic materials. A wide range of research are in progress to investigate the possibilities of creating internal magnet for magnetic actuators. With a microfabrication technique, 10 turns of micro magnetic coil are made of nickel-coated plate and polysilicon torsion bar [136]. The micromirror is attached to the torsion bar and can be manipulated by an out-of-plane excursion and off-chip magnetic field. With a current flow of 500 mA, the device can deflect up to 45° out-of-plane. Several materials are also examined for a magnetic coil used in microfabrication process as well [137]. Jun *et al.* reported a pattern of copper coil for a high-speed and large-scale electromagnetically actuated resonant MEMS optical scanner [138]. The device has one possible rotation mode with the micromirror area of 6 mm × 4 mm. The maximum optical deflection angle of ±6.8° at 2.95 kHz resonant frequency. Yang *et al.* demonstrated the copper micromirror that is driven by the eddy-current-induced Lorentz force, whereas the ferromagnetic (electroplated Nickel) micromirror is mainly driven by the magnetostatic force [139]. The optical scanning angle of this device is 20° at an input power of 9 mW and is capable for two-dimensional scanning patterns. Electromagnetic micro-actuator arrays can be made of CoPt planar coil for a thickness of 5–10 μm, however, the maximum deflection is only 1.2 μm [140]. Techniques on micromachined coil are also used in a silicon-lithography-electroforming with a frame of 12 mm × 24 mm and Au electroplating coil [141]. The device has a maximum deflection angle of 9° at 1311 Hz. Moreover, the miniaturization of micro magnetic induction machines is designed for portable application with a micromachined of 1-mm-thick NiFe wafer for a non-laminar stator [142]. A method of pressing between Lithography, Electroplating, and Molding of Polymethylmethacrylate (LIGA PMMA) mold and NdFeB power composite is also alternative for forming a structure [143]. This method can create a permanent magnet with a 5 μm feature size and 200 μm height. Nevertheless, silicon carbide and BCB polymer is also investigated for a rotary micromotor with microball bearing [144].

### 2.5. Other Actuators

In addition to the described actuators, other types of actuators were discovered for scanning micromirrors as well. Shape memory alloy is one of these alternative micro-actuators for scanning micromirrors [145]. Examples of shape memory alloy for scanning micromirrors were demonstrated by Fu *et al.* This design of micromirror structure is formed by sputtering TiNi shape memory thin films [146]. With a 3.5-μm-thick-TiNi cantilever on silicon membrane, the micromirror can achieve up to 190 μm vertical displacement at 5 V. With this design, the maximum optical angle is 6°–10°. In addition, Haga *et al.* reported a miniature pressure sensor for imaging intravascular of human body. In this study, TiNi shape memory alloy (SMA) microcoils were fabricated by photolithography and patterned by etching processes to create an active catheters and guild wires of the device as well [147].

Pneumatic actuators is another choice of micro-actuator for micromirror. Pressure can also manipulate the deformation and displacement of micromirror in several ways. For example, Werber *et al.* reported a tunable pneumatic micromirror that is embedded on a 50-μm-thick PDMS. The maximum angle of 75° at 65 kPa [148,149]. When combining with thermal actuators, the same group of researchers reported a thermo-pneumatically actuated membrane-based micromirror. The maximum tilting angle of the micromirror is 13° at 30 V (310 °C—temperature). With a formation of seven hexagons of heating locations, the stage can move up to 80 μm for a piston motion [150].

**Figure 5:**
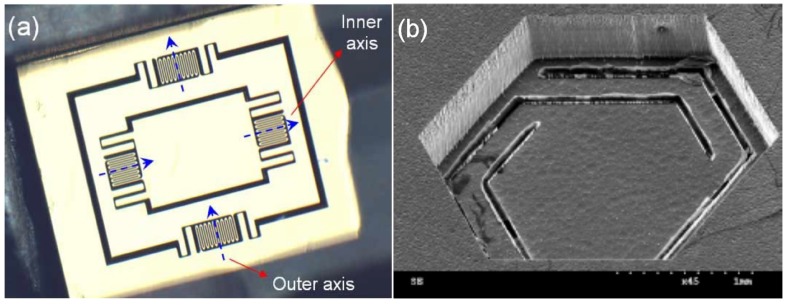
Alternative choices for scanning micromirror with (**a**) magnetic actuators by Kim *et al.* [129], and (**b**) shape memory alloy by Fu *et al.* [146]. Reproduced with permission from [129,146].

The summary on scanning micromirrors with other type of actuators are shown in Table 4. An example of magnetically-driven scanning micromirrors is shown in Figure 5a. Morevoer, a sample of shap memory alloy is shown in Figure 5b.

**Table 4:** Relevant work on micromirrors with other actuators.

Actuation	Reference	Year	Size (in mm)	Characteristics	Operating Conditions	Natural Frequency
Electromagnetic	Cho *et al.* [134]	2002	0.8 dia. mirror	±4.35°, ±15.7°	4.2 V/1.7 V	-
Electromagnetic	Ahn *et al.* [133]	2004	3.5 × 3.5 mirror	±1.51°, ±5.71°	20 mA	920 Hz
Electromagnetic	Mitsui *et al.* [135]	2006	7.4 × 9.8	±8° static	4.6 mA	80.5 Hz
Electromagnetic	Kim *et al.* [129]	2007	2.4 × 2.9	±20° optical	3V	350 Hz
Electromagnetic	Gokdel *et al.* [130]	2009	-	11.7°, 23.2°	42 mW	350 Hz
Pneumatic	Werber *et al.* [148]	2006	2.2 × 2.5	40°	30 kPa	-
Thermo-Pn	Werber *et al.* [150]	2006	2.2 × 2.5	13°	30 V	10 mHz
			11 × 11	80 μm	20 V	-
Shape Memory	Fu *et al.* [146]	2005	2.2 × 2.2	190 μm (15°)	5 V, 30 mA	0.1 Hz

## 3. Discussion

The designs of scanning micromirrors based on MEMS technology are widely investigated for biomedical applications. A suitable design are depended on targeted applications and several parameters, such as size, range of motion, scanning speed, operating voltage, actuator type, and integration processes. These parameters affect the characteristics of MEMS scanning micromirrors in different ways and it can be explained in the following aspects.

### 3.1. Target Application

With the processes on MEMS/BioMEMS technology, surface micromachining, and selective etching process of substrates, various microscale devices with different mechanisms and designs are fabricated with high precision and high resolution. These processes are attractive for biomedical device because of smaller size, performances, and integration processes. Current research in scanning micromirrors focuses on implementation of micromirrors for endoscopy and OCT. Optical imaging with scanning micromirrors are reported for higher sensitivity, lower light fluence rate, higher speed, and higher resolution. The resolution of OCT is about 2.4 μm and 10 μm for side-imaging and forward-imaging endoscopic OCT. This characteristic is outstanding, compared to conventional methods of imaging such as endoscopic ultrasonography, needle biopsy, electron beam CT, PET, ultrasound, mammography, and MRI. With a development on OCT, medical processes for diagnosis and early detection of diseases can be improve for quality, accessibility, and speed. Examples of bioimaging diagnostics that can be implemented with OCT include gastrointestinal (GI) tract, intravascular system (coronary artery disease), respiratory tract (sleep apnea, laryngeal carcinoma, bronchial inflammation, gynecologic cancers, prostate cancer, and urinary bladder cancer, ovary and uterus tissue, breast cancer and liver biopsies, brain-related disorders (hydrocephalus, cerebral aneurysms) and brain tumors.

### 3.2. Size

For scanning micromirror, shape and configuration are important parameters that influence performances of endoscopic devices. In general, smaller size will increase the performances of the scanning micromirrors because of smaller weight, less stiffness, and larger natural frequency. Material thickness is also important for the characteristics of scanning micromirrors. Thickness of actuators can affect the performances in two different ways. For electrostatic actuators, thicker comb configurations will increase electrostatic force, but will not affect directly on the deformation of scanning micromirrors because it will cancel to the stiffness of the flexure spring suspension. For other types of micro-actuators, thinner membranes will increase the performances of the scanning micromirror because of the deformation of the actuator can be increased. However, thin membranes and mirrors can be weak for handling and assembling.

The device dimension of scanning micromirrors is a crucial parameter for OCT and biomedical applications. In order to perform minimally invasive endoscopic imaging, scanning micromirror are required to be compact. Typical dimensions of MEMS scanning micromirror ranges from 0.3 μm to 10 mm. However, the overall dimensions that are less than 5 millimeters are preferred for general endoscopic systems. Size of reflected mirror is also important parameter especially for laser surgery. It is also noted that the micromirror dimension should be larger than laser spot size as well.

### 3.3. Range of Motion

**Figure 6:**
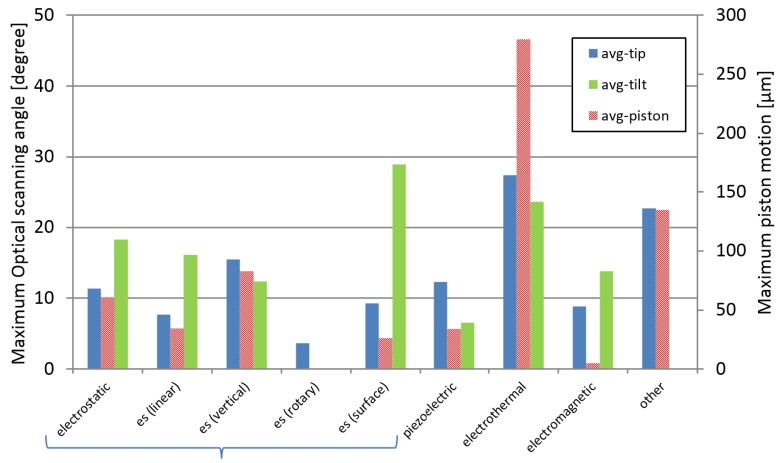
Comparison on performances for all types of MEMS micro-actuators in scanning micromirrors. The area with sloped-lines represents the averaged value for piston motion. The shaded area represents the average value for rotational angles.

**Figure 7:**
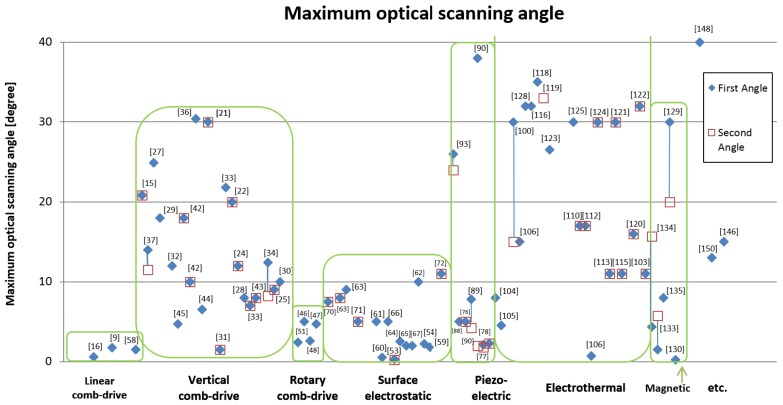
Scanning micromirror performances for angular motions. A number in square rectangle is the reference number. The *X*-axis shows group of actuator types, ranging by year of publications. The *Y*-axis shows the averages values for the maximum optical scanning angles.

**Figure 8:**
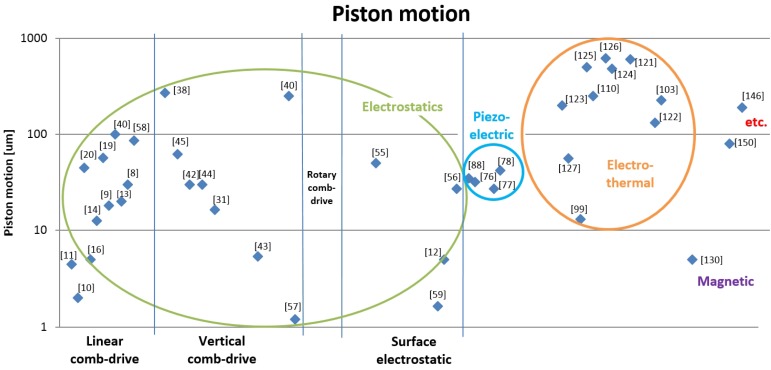
Scanning micromirror performances for translational motions. A number in square rectangle is the reference number. The *X*-axis shows group of actuator types, ranging by year of publications. The *Y*-axis shows the values for the maximum displacements in piston motion.

In Figure 6, the average values of micromirror performance for all type of actuators are compared. The performances on maximum optical scanning angle for each type of actuators is shown in Figure 7. Moreover, the values of maximum displacement on piston motion for all type of micromirrors are compared in Figure 8. For the recent development, the average performances of electrostatic actuators are about 12° and 60 μm. Considering each subgroup, the average angular and transverse motion of the vertical comb actuators are higher than that of linear comb-drives, rotary comb-drives, and surface electrostatic. With the average motion of 16° and 80 μm, vertical comb actuators are very attractive for research and development of scanning micromirrors. It is also noted that the rotary comb-drives cannot generate any out-of-plane motion for scanning micromirrors. For linear comb actuators, the maximum range rotation is up to 20° with the translation distance up to 60 μm. The range of natural frequency is between 300–5000 Hz with a maximum operating voltage of 200 V. Among all designs in electrostatic actuators, the maximum value of rotational range is 46° and the maximum piston motion 270 μm that can be achieved by vertical comb-drives. The range of natural frequency for vertical comb actuator is between 150–34,000 Hz with a maximum operating voltage of 250 V. The rotary comb is capable for one rotational movement with the maximum rotation angle of 4.7° at 70 V. Moreover, the parallel-plate actuators can perform up to 5° mechanical angle with a small motion in piston direction. The range of natural frequency for parallel-plate actuators is between 50–59,000 Hz with a maximum operating voltage of 300 V. It is noted that the operating voltage for biomedical devices should not be high. Although some scanning micromirrors are reported for a high performance, they might damage tissues or living cells if the operating voltage is high or the electrical circuit is shorted. The recommended range of voltage for biomedical devices is about 100 V.

For the scanning micromirrors with piezoelectric actuators, the average performances are at 12° and 34 μm for angular motion and out-of-plane motion, respectively. Piezoelectric actuators can perform up to 25° in two axes. The piston motion of piezoelectric actuators can be up to 180 μm with the operating voltage up to 20 V. For the scanning micromirrors with magnetic actuators, the average performances are at 15° and 5 μm for angular motion and out-of-plane motion, respectively.

Among all type of scanning micromirrors, the electrothermal actuators are reported for the best performances in both rotational motions and out-of-plane motion. The average of angular motion are about 27° and 280 μm for piston motion. For electrothermal actuators, the performances of scanning micromirrors can be up to 35° in two axes. The piston motion for electrothermal actuators can be up to 600 μm with the operating voltage up to 50 V. An advantage of electrothermal actuators is initial elevation due to residual stress of material after microfabrication process. Some designs demonstrated an initial elevation of the micromirror platform up to 300 μm. While the searching for better scanning micromirrors, difficulties to fabricate micromirror with multi-DOFs is more than those with a single DOF. It is also observed that for the single-DOF platform, the average of optical scanning angles is larger than the multi-DOF platform.

### 3.4. Scanning Speed

**Figure 9:**
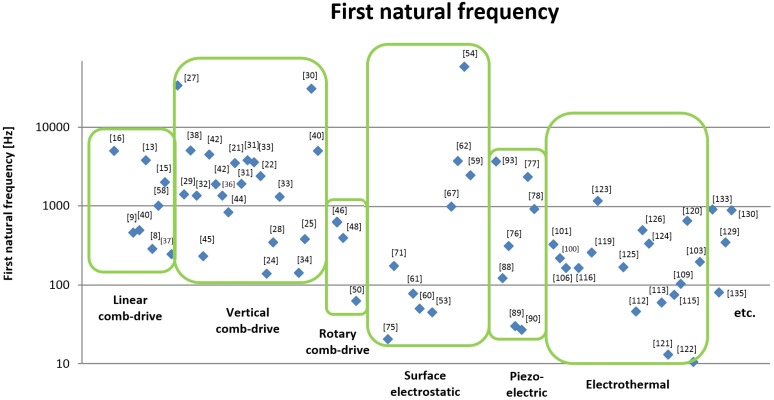
Scanning micromirror performances based on their first natural frequency. A number in square rectangle is the reference number. The *X*-axis shows group of actuator types, ranging by year of publications.

In Figure 9, the first natural frequency of all micromirrors are plotted. It is observed that the average natural frequency of the electrothermal micromirrors is in the range of 10–1000s Hz, while the average natural frequency of the comb-drives is in the range of 100–10,000s Hz. For piezoelectric actuators, the average natural frequency is in the range of 10–1000s Hz. The response time for dynamic system is about 5 ms for scanning micromirrors implemented in OCT devices.

### 3.5. Operating Conditions

Operating conditions are also important parameters for testing and implementing the scanning micromirrors. From literature review, several research methodologies and testing devices are used for characterization of MEMS scanning micromirrors. It can be observed that the operating conditions of scanning mirrors can be varied from static responses, dynamic responses, and near-singularity responses. Although all of operating conditions can be controlled with a complex control scheme, the implementations of resonance frequency is more difficult than those of static modes. Due to different behaviors of scanning micromirrors at different frequencies, specific requirements are necessarily considered for implementing micromirrors in biomedical applications.

### 3.6. Summary

To summarize the advantages and disadvantages for each type of actuators, Table 5 shows the outstanding characteristics of micro-actuators for scanning micromirrors. Challenges for development on electrostatic actuator is pull-in voltage and high driving voltage. Piezoelectric actuators also have issues of charge leakage and hysteresis of materials. For electrothermal and electromagnetic actuators, power consumption is a problem. Moreover, electromagnetic actuators require external magnets that can be large in size and can create electromagnetic interference.

**Table 5:** Comparisons of micro-actuators for medical scanning mirrors.

Actuation type	Advantages	Disadvantages
	Fast response	Pull-in effect
**Electrostatic**	Low power consumption	High driving voltage
	Large scan angle	
	Larger driving force	High power consumption
**Electromagnetic**	Lower driving voltage	External magnets
	Large scan angle	Electromagnetic interference
	Fast responses	Large initial tilting angle
**Piezoelectric**	Large bandwidth	Charge leakage problems
	Low power consumption	Hysteresis effect
	Large scan angle	High power consumption
**Electrothermal**	Low driving voltage	Slow response
	High fill-factor	

Because each type of actuators has advantages and disadvantages, the alternative choices for future development are the integrations of various types of actuators. In the literature, several studies focus on combining various types of micro-actuators. Examples of this trend are demonstrated by Zhang *et al.* for integrated design of thermal actuator with comb-drive electrode for capacitive sensing. This device can achieve an out-of-plane displacement of 24 μm at 17 mW, thermal time constant of 0.24 ms, a mechanical resonant frequency of 16.8 kHz [151]. Moreover, Coa *et al.* demonstrated an integration of electrothermal and electromagnetic actuator by using a polyMUMPs on SOI wafers [152]. The device is reported for a displacement of 120 μm at 20 Hz.

## 4. Future Work

Nevertheless, the development of scanning micromirrors for biomedical application is still attractive for engineers and scientists. There are some projects that aim to facilitate the surgery by using scanning micromirrors. Our current project funded by the European Commission’s 7th Framework Program also focuses on cognitive and robotic systems operating in real-world environments. This is an integrative project between several research institutes and clinical laboratories for investigation scanning micromirrors for laser phonomicrosurgery applications. This system consists of a flexible endoscope with an actuated mirror, a stereo-vision and high speed visual servoing, and an augmented reality man-machine interface for assisted teleoperation. (www.microralp.eu) [153,154]. Moreover, there are currently some commercial products of scanning micromirrors that can connect to different applications. This also emphasize the importance of scanning micromirrors for future developments [155,156].

## 5. Conclusions

Principles of actuation and recent developments that are presented in this paper can assist researchers and scientists for designing. However, the completed system should be evaluated and collaborated with the optics, visions, mechatronics, cognition, and power. With a development of scanning micromirrors for medical applications, capabilities of microsurgery apparatus will be enhanced for better safety, quality of surgical procedures, accessibility of the endoscopic system, dexterity of surgeons, manipulation skills of surgeon as well. According to the types of actuators, scanning micromirrors are studied with electrothermal, electrostatic, piezoelectric, magnetic, shape memory alloy, and pneumatic actuators.

Among the described actuators, electrothermal actuators are widely studied for medical applications. However, the challenges for electrothermal actuators are speed, charge leakage, and hysteresis effect. These issues are investigated and developed by studying of kinematic and control. However, there are still potential actuators that can be used for medical applications, such as electrostatic actuators, and piezoelectric actuators. The combination of more than one type of actuators is also alternative as well.
